# Supplementary material for: Ancient Clostridium DNA and variants of tetanus neurotoxins associated with human archaeological remains
Source: Nat Commun. 2023 Sep 6;14:5475. doi: 10.1038/s41467-023-41174-0 (PMC10482840; doi:10.1038/s41467-023-41174-0)
Supplement: Supplementary file 1 — Supplementary Information [file 41467_2023_41174_MOESM1_ESM.pdf]

Supplementary Information

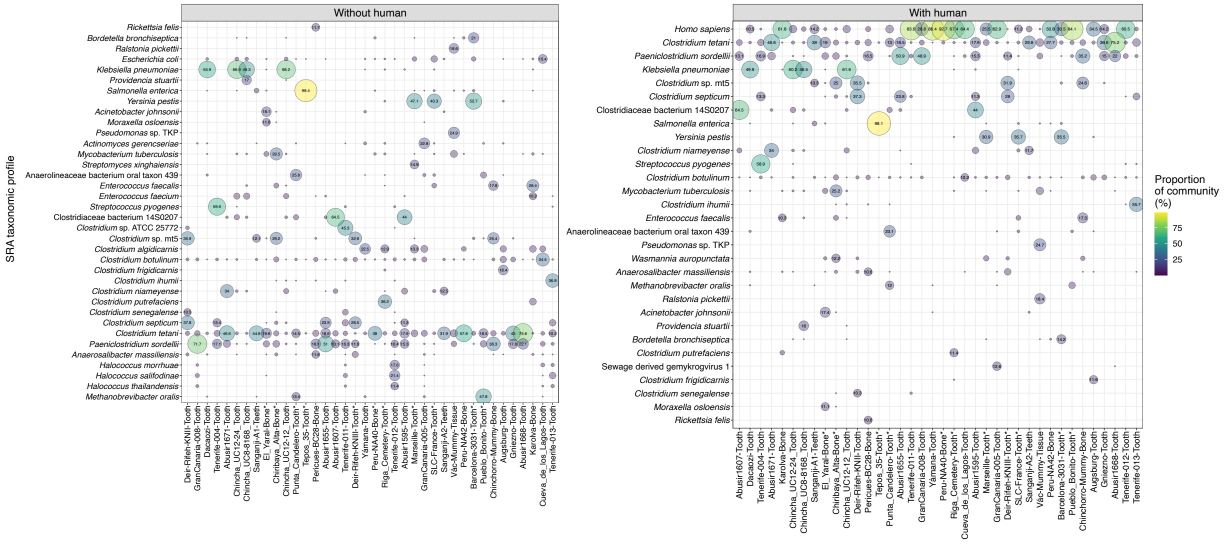

Supplementary Figure 1. Proportional abundance of taxa detected in metagenomes from 38 aDNA samples (microbial only – left, all – right). Abundance values are based on NCBI sequence read archive taxonomic profiles including all identified bacterial and archaeal species. Only species with >10% abundance in at least one sample have been plotted, and the full dataset is available in Supplementary Data 3. Only values greater than 10% are labelled in the figure. For BioSamples associated with more than one SRA ID, the sample with a median count of *Clostridium tetani* was chosen. In the case of a choice between two, a random SRA ID was chosen. The samples here are represented as follows: Sanganji-A1-Teeth: DRR046398; Sanganji-A2-Teeth: DRR046408; Augsburg-Tooth: ERR2112574; GranCanaria-005-Tooth: ERR2111951; GranCanaria-008-Tooth: ERR2112080; Chinchorro-Mummy-Bone: ERR966303; SLC-France-Tooth: ERR2862150; Peru-NA42-Bone: SRR1298752; Pueblo_Bonito-Tooth; SRR5169887. *Sample names with asterisks indicate those associated with capture sequencing methods. Source data are provided as a Source Data file.

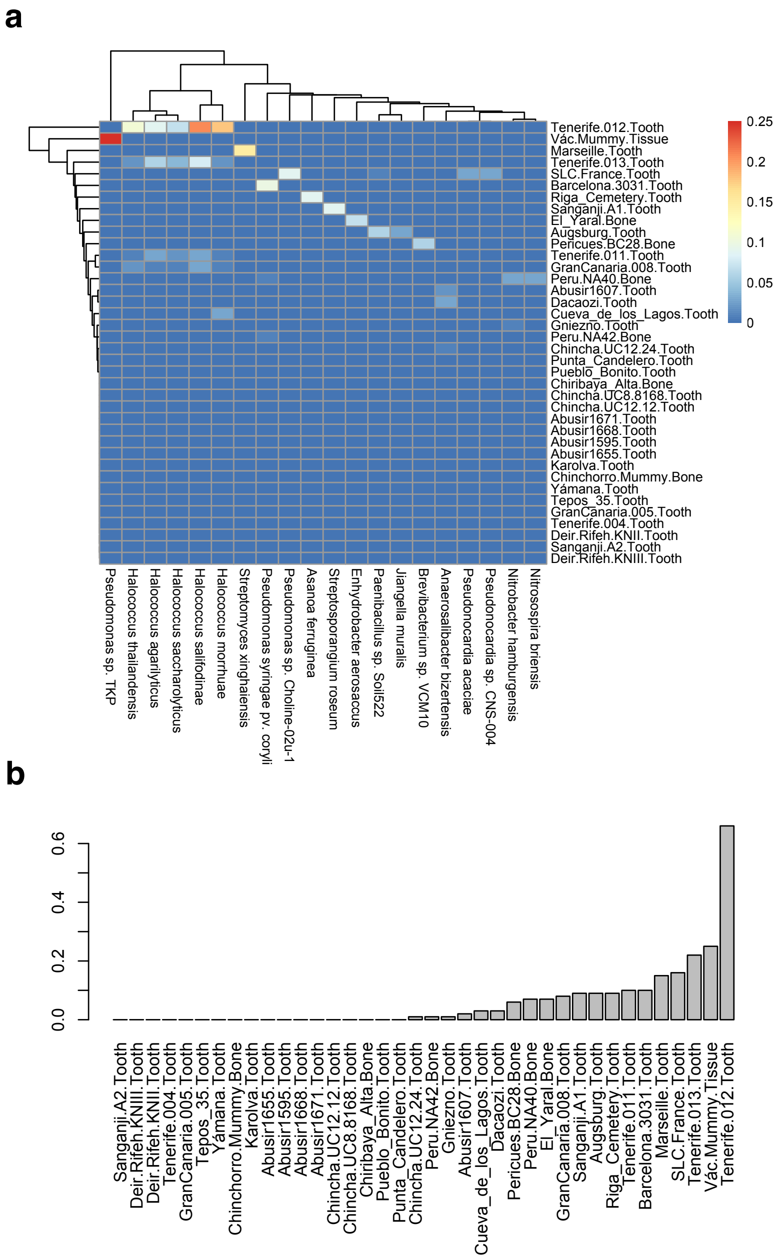

Supplementary Figure 2. Estimated abundance of environment-specific microbial taxa in 38 aDNA samples. (**a**) Relative abundance heatmap of environment-specific microbes for each dataset. (**b**) Total estimated abundance of environment-specific microbes for each dataset. Environment-specific microbes were defined as shown in Supplementary Data 4. The total abundance of environment-specific microbes was calculated for each dataset (Supplementary Data 4) by summing their individual abundance profiles based on the STAT analysis in Supplementary Data 3. Source data are provided as a Source Data file.

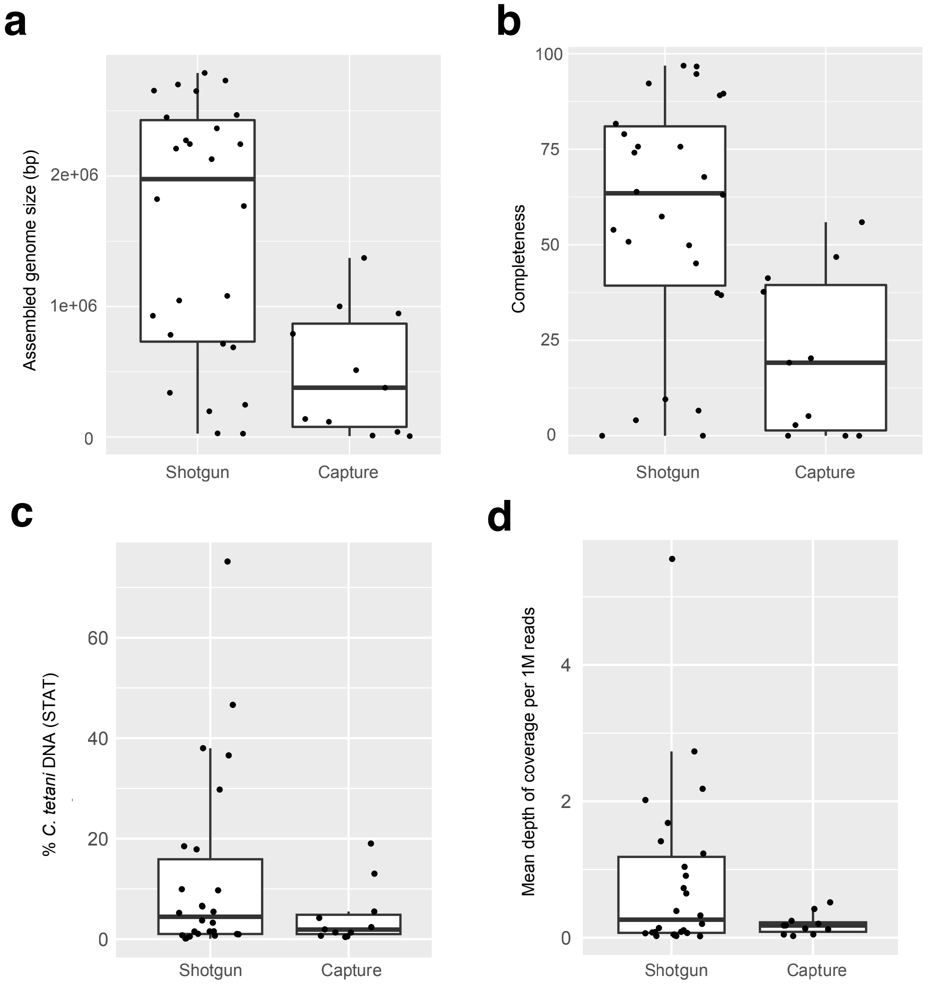

Supplementary Figure 3. Assembly size and completeness of recovered acBins based on use of shotgun versus capture sequencing methods. The data analyzed include n = 37 biologically independent samples (n = 11 from capture datasets, n = 26 from shotgun datasets). Peru-NA42-Bone was not included in (a-d) as it contains a mix of pre- and post-capture sequences. The assembly size (**a**) and completeness (**b**) statistics were calculated using CheckM and are available in Supplementary Data 8. The % of species-classified *C. tetani* DNA (**c**) was derived from the STAT data in Supplementary Data 3. (**d**) is derived by calculating the average depth of coverage of reads mapped to the *C. tetani* reference chromosome (see Supplementary Data 17), and normalizing this value to the total number of reads (see Supplementary Data 22). The boxplots in (a-d) depict the lower quartile, median, and upper quartile of the data, with whiskers extending to 1.5 times the interquartile range (IQR) above the third quartile or below the first quartile. Source data are provided as a Source Data file.

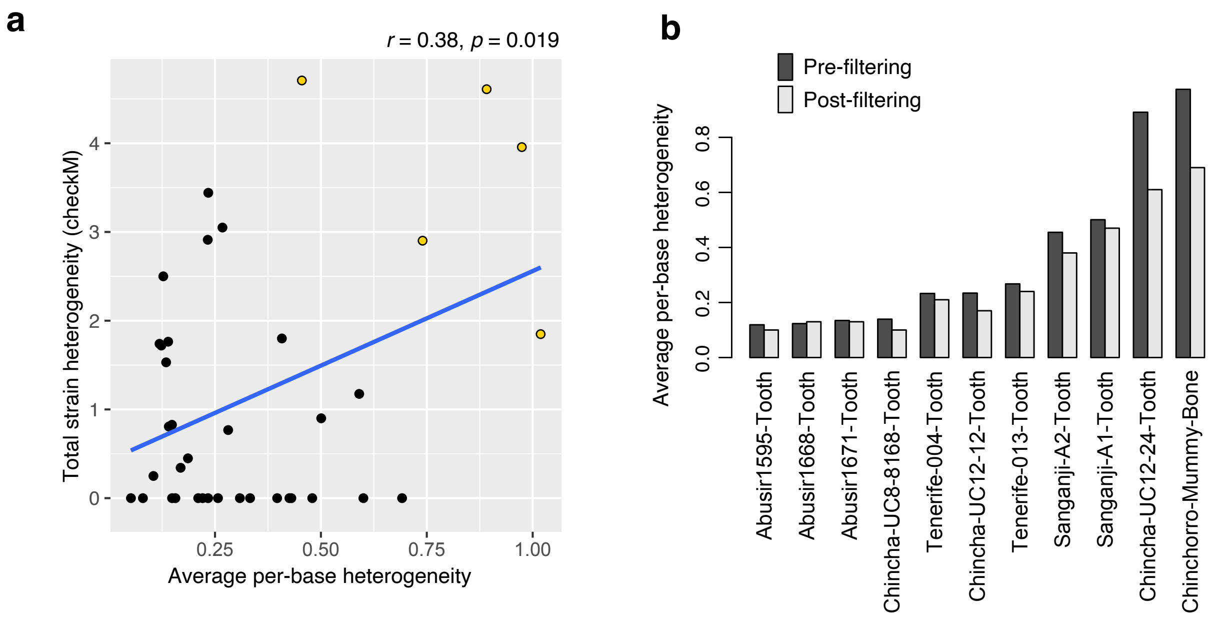

Supplementary Figure 4. Comparison of strain heterogeneity levels estimated from mapped base heterogeneity versus CheckM calculations. (**a**) Average per-base heterogeneity (x-axis) was calculated for each acBin by examining mapped reads for all positions across contigs, and calculating the fraction of bases that disagree with the reference base. We then calculated the mean of the per-base fractional heterogeneity value across all non-zero coverage positions. Total strain heterogeneity was calculated as the product of CheckM contamination and the strain heterogeneity as measured using CheckM. The five samples with the highest probability of containing strain variation are shown as yellow circles. These include: Sanganji-A2-Tooth, Chinchorro-Mummy-Bone, SLC-France-Tooth, Karolva-Tooth, Chincha-UC12-24-Tooth. A Pearson correlation coefficient was calculated with an associated *p*-value (two-sided test, based on t-distribution, no adjustment for multiple comparisons). (**b**) Mapped base heterogeneity of 11 acBins following further filtering of reads and samples. Contigs were further filtered to only include those that mapped to the final recombination-filtered Parsnp alignment used in Fig. 2c. Reads with mapping quality < 30 were removed, and sites with extreme coverage (>3x standard deviation from mean coverage) were also removed. As shown in (**b**), heterogeneity values were reduced following these filters. Source data are provided as a Source Data file.

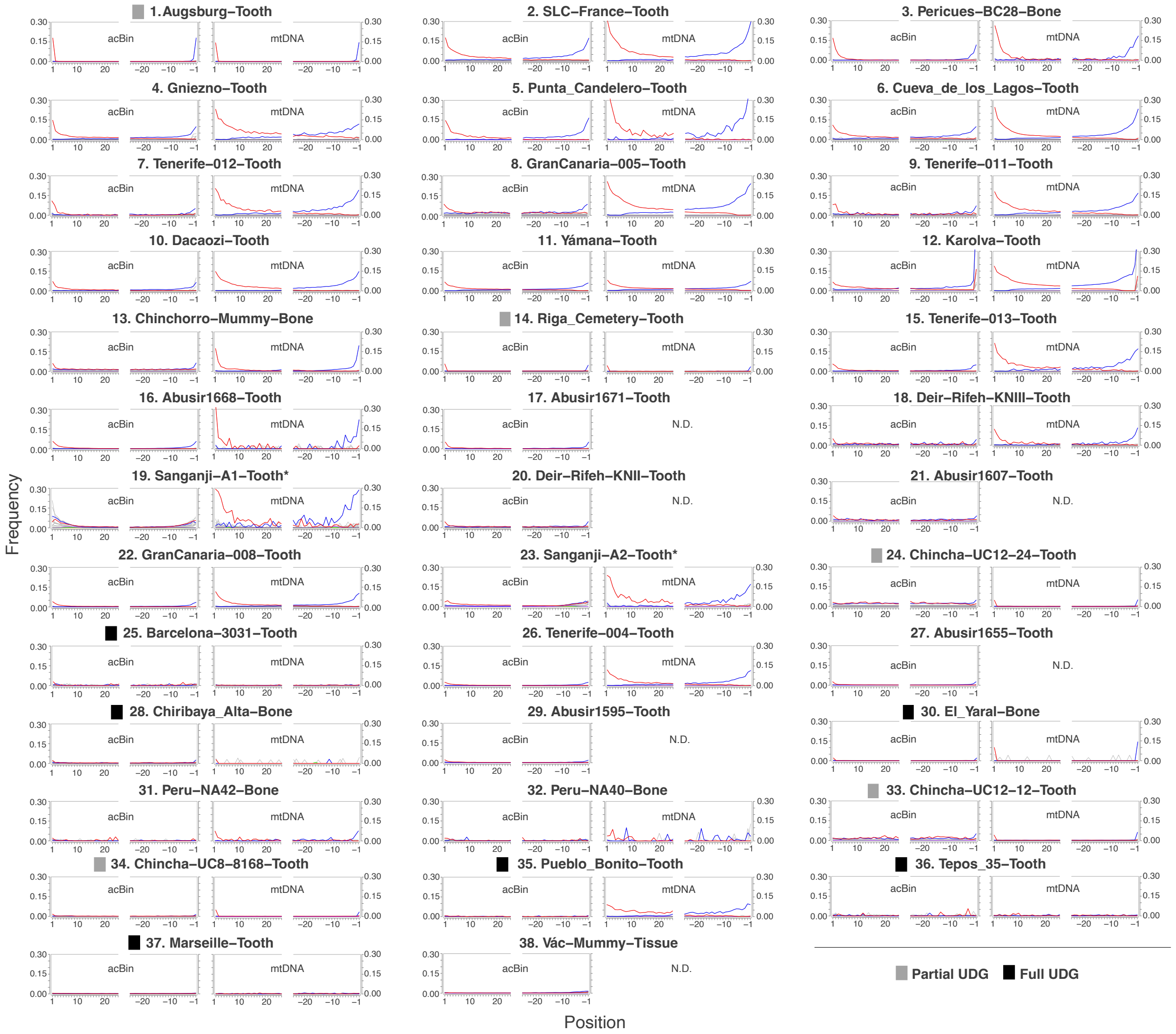

Supplementary Figure 5. MapDamage profiles of acBin DNA and human mtDNA fragments from 38 ancient DNA samples. Samples are numbered in order of decreasing acBin 5' C→T misincorporation rates. G→A misincorporations (blue); C→T misincorporations (red). Many ancient samples show a characteristic pattern of increased C→T misincorporations at the 5' end and complementary G→A mutations at the 3' end of sequence fragments. mtDNA mapDamage profiles for six samples (labeled "N.D" for not detected) were omitted as the number of reads mapping to human mtDNA was too low (below 50 reads) for analysis. *Two samples (Sanganji-A1-Tooth and Sanganji-A2-Tooth) are notable as they have an elevated rate of other mutations (gray) at the edges of reads. Manual inspection of these samples revealed problematic read alignments to contigs involving short non-adaptor sequence fragments at the edges of a small proportion of reads. Source data are provided as a Source Data file.

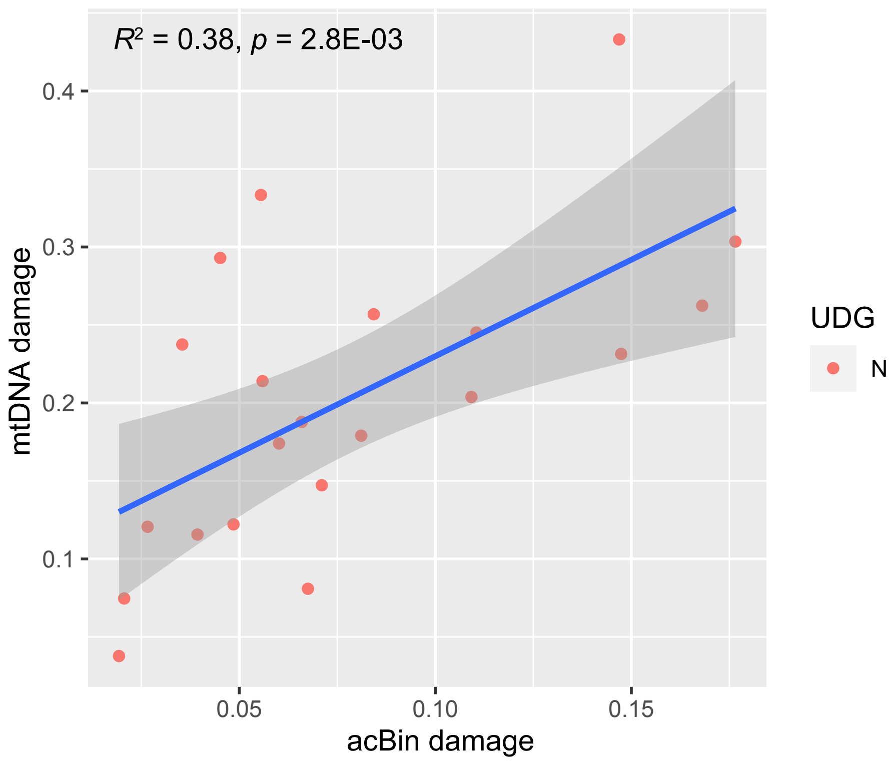

Supplementary Figure 6. Correlation between damage rates of acBins and corresponding human mtDNA from the same sample. A Pearson correlation coefficient was calculated with an associated *p*-value (two-sided test, based on t-distribution, no adjustment for multiple comparisons). Also shown are 95% confidence bands computed using a linear smoothing function in ggplot2. The acBins are also colored based on the UDG treatment used in the original aDNA samples. The damage rates are the 5' first position C→T misincorporation rate determined using mapDamage. Raw data is available in Supplementary Data 2 and Supplementary Data 9. Six samples were removed as the number of human reads was insufficient to calculate mtDNA damage profiles (see Supplementary Figure 5). Source data are provided as a Source Data file.

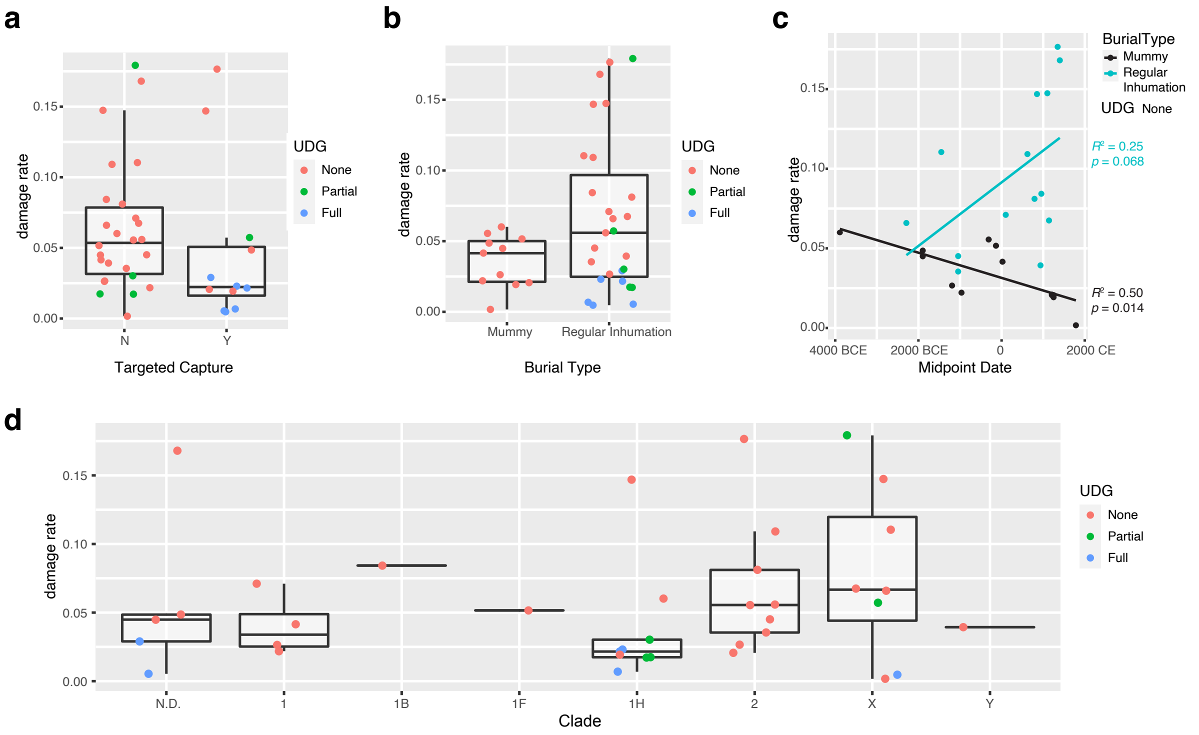

Supplementary Figure 7. Exploration of acBin damage levels versus various sample metadata. The data analyzed here include n = 38 biologically independent samples subdivided by different metadata categories. Damage levels are plotted as a function of: (**a**) whether capture methods were used (n = 26 without capture, n = 12 with capture); (**b**) burial method (n = 11 mummy, n = 27 regular inhumation); (**c**) midpoint date of archaeological sample (trendlines shown with Pearson correlation coefficients, two-sided tests, based on t-distribution, no adjustment for multiple comparisons); (**d**) phylogenetic clade (n = 5 N.D., n = 4 from clade 1, n = 1 from clade 1B, n = 1 from clade 1F, n = 9 from 1H, n = 9 from clade 2, n = 8 from clade X, n = 1 from lineage Y). In d, the "N.D." group includes low coverage acBins that could not be phylogenetically placed. For plots in a, b, and d, samples are also colored according to their UDG status, as it is a main factor that influences the damage level. For the plot in c, samples with full or partial UDG treatment were removed as this is known to affect damage rates. The boxplots shown in a, b, and d depict the lower quartile, median, and upper quartile of the data, with whiskers extending to 1.5 times the interquartile range (IQR) above the third quartile or below the first quartile. Source data are provided as a Source Data file.

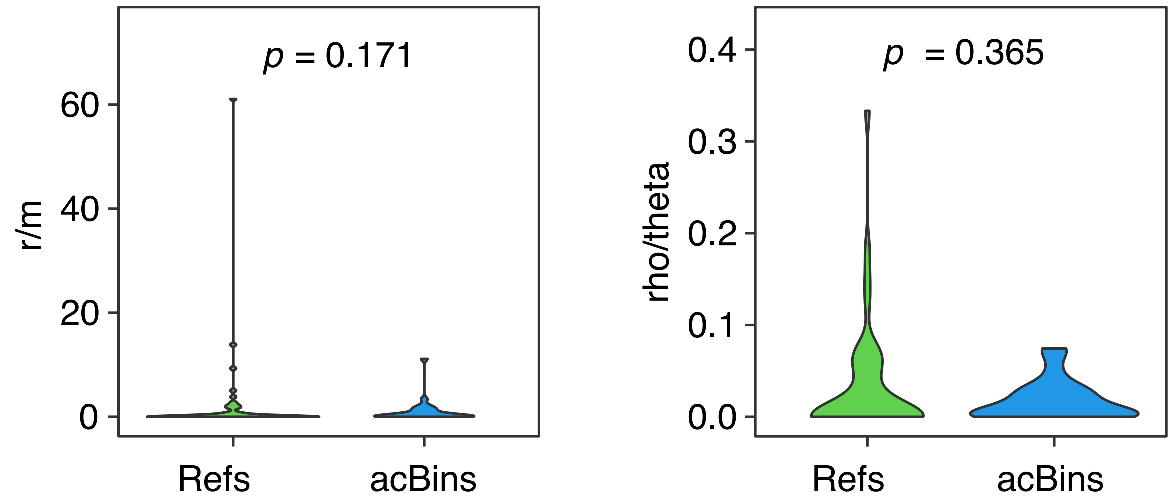

Supplementary Figure 8. Analysis of recombination using Gubbins. Comparison of estimated recombination levels between acBins and modern strains reveals no significant differences (two-tailed t-test, no adjustment for multiple comparisons). The r/m ratio is the ratio of the probability that a site was altered by recombination (r) and mutation (m). The rho/theta ratio measures the rate of recombination (rho) relative to the rate of mutation (theta). Genomes with gap content of 90% or greater were excluded. Source data are provided as a Source Data file.

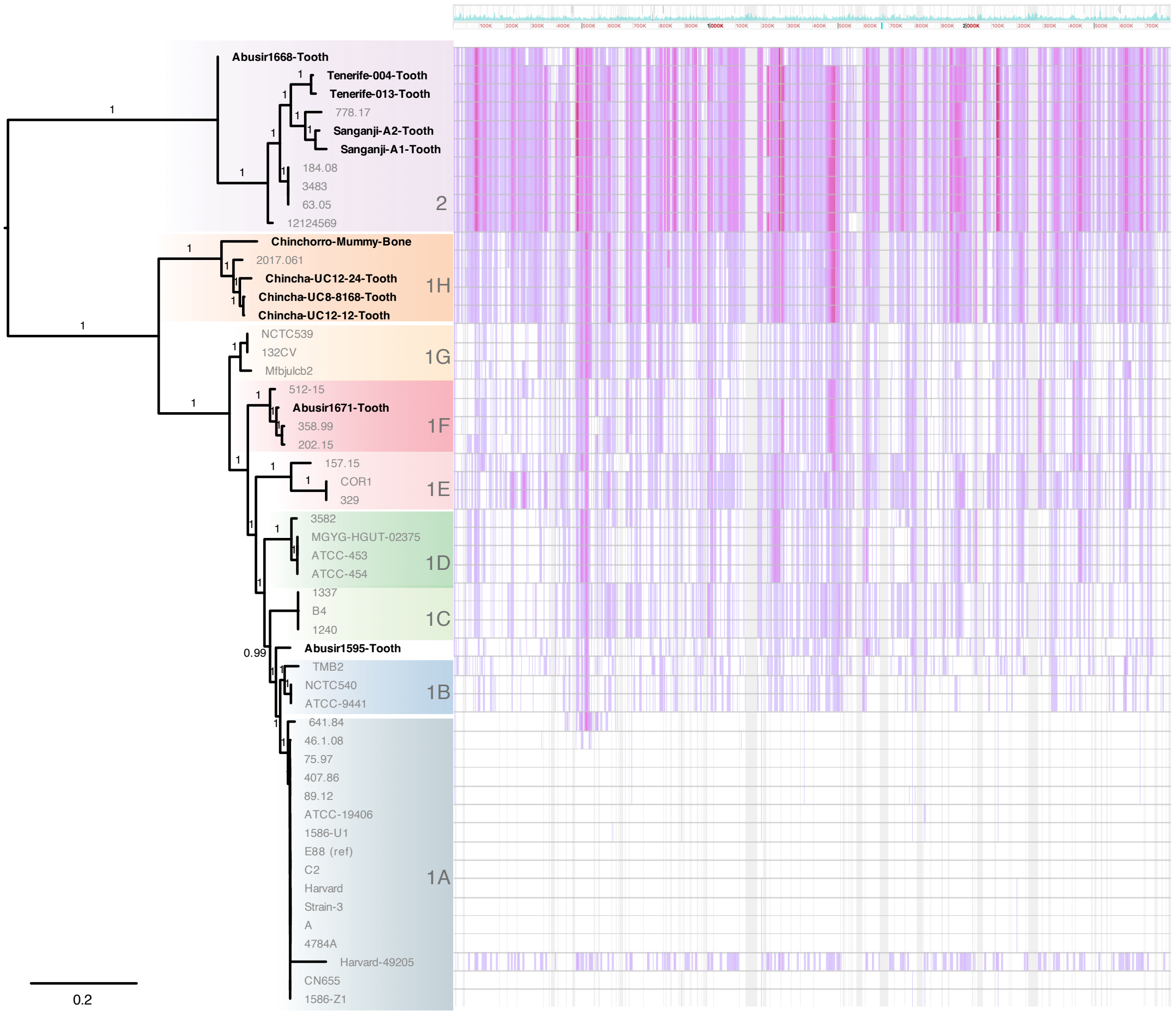

Supplementary Figure 9. SNP-based phylogenetic tree of a reduced set of acBins from *C. tetani* lineages 1 and 2. The tree was generated using Parsnp and only the acBins that passed Parsnp's default parameters are included. The phylogenetic tree, labeled by *C. tetani* lineage, is shown on the left with acBins shown in bold text. A visualization of the corresponding alignment using the Gingr tool is shown on the right relative to the E88 chromosome, with the SNP density visualized as a gradient of light to dark purple colored lines. Here, SNPs are defined relative to the E88 reference genome, which is located in lineage 1A. Clade support (aLRT) values are indicated above the nodes in the tree. Source data are provided as a Source Data file.

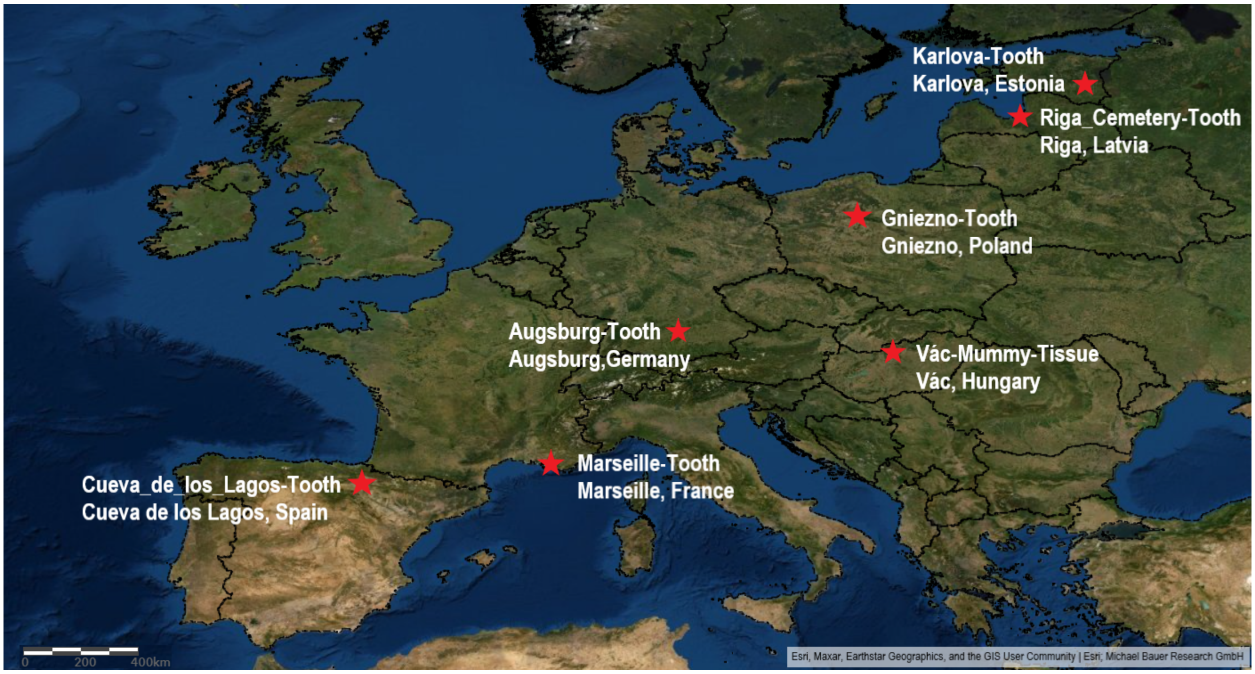

Supplementary Figure 10. Geographic location of 7/8 archaeological samples associated with *Clostridium* sp. X. This map was made by the authors using ArcGIS online, with the World Imagery (WGS84) basemap credited to: Esri, Maxar, Earthstar Geographics, and the GIS User Community; as well as the Europe_Countries_2017 layer, credited to: Esri, and Michael Bauer Research GmbH.

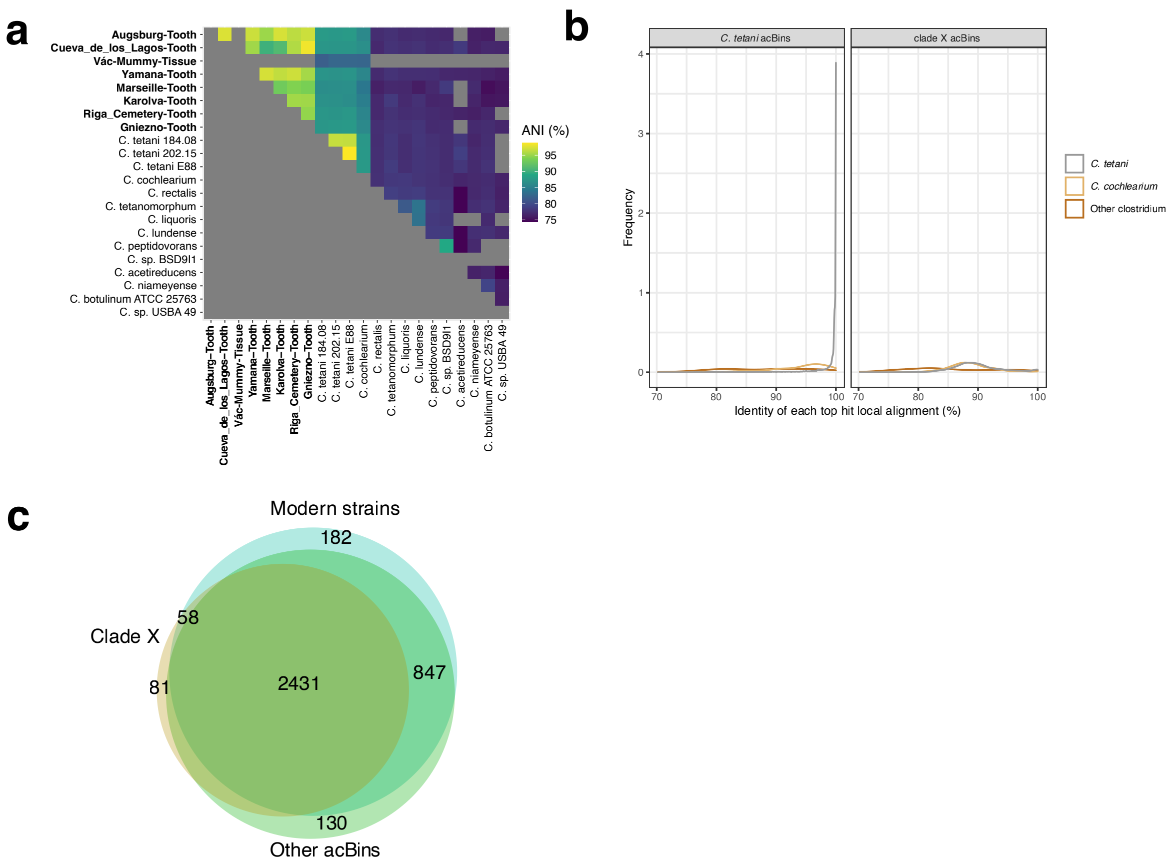

Supplementary Figure 11. Sequence similarity levels between clade X acBins and reference *Clostridium* genomes. (**a**) Pairwise genome-wide average nucleotide identities (ANIs) involving acBins, representative *C. tetani* genomes, and other genomes from *Clostridium* species. (**b**) Density plot of sequence identities in local alignments between acBins and reference genomes (*C. tetani*, *C. cochlearium*, other *Clostridium* species), subdivided by clade X acBins and non-clade X acBins. (**c**) Venn diagram indicating overlap of predicted orthogroups between acBins, clade X, and modern *C. tetani* genomes. Source data are provided as a Source Data file.

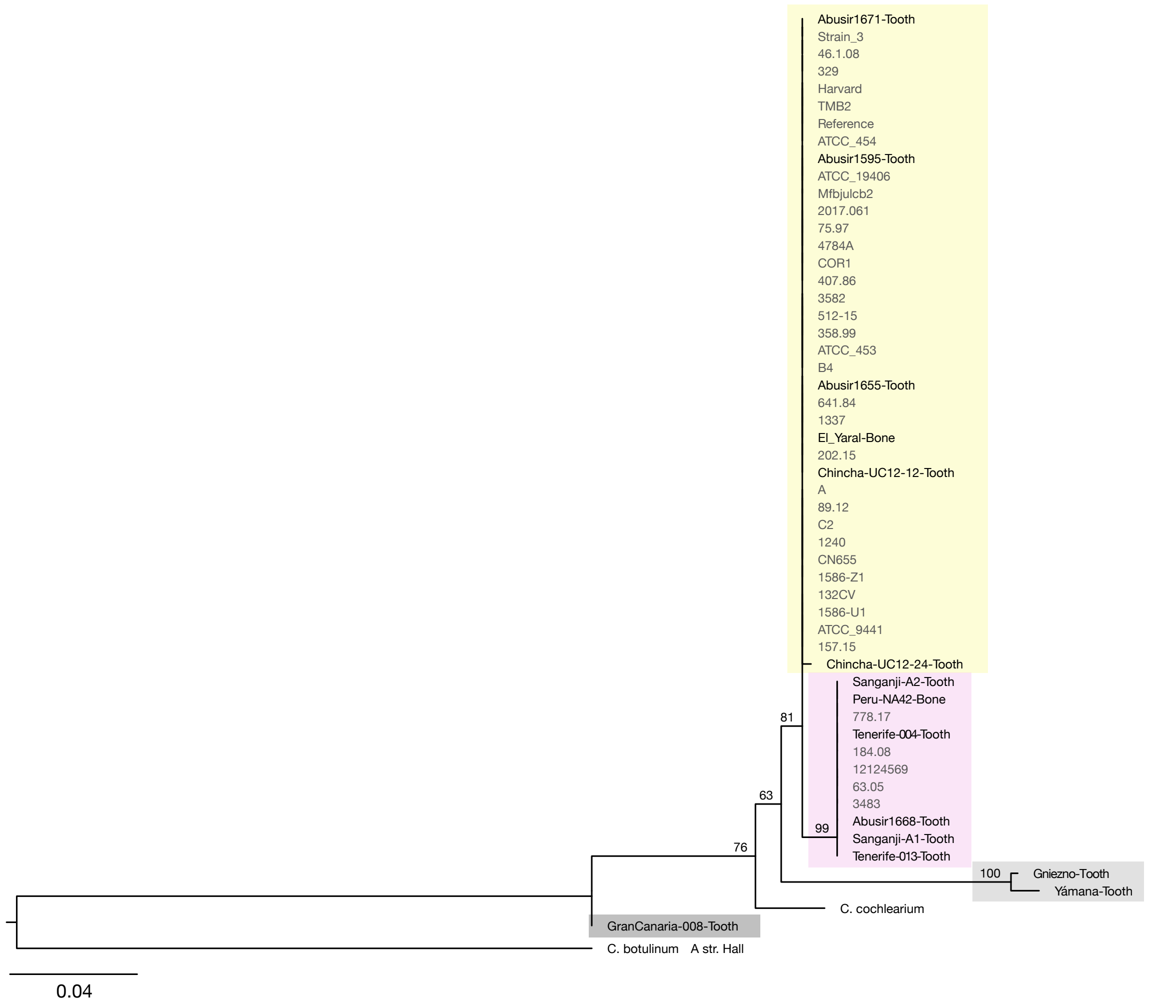

Supplementary Figure 12. Phylogenetic tree of *rpsL* coding sequences. Trees are based on a blastn search with *Clostridium tetani* E88 sequences. The phylogeny is based on a multiple alignment of *rpsL* (AE015927.1:c2752816-2752442) sequences identifed from aDNA *C. tetani* contigs and modern *C. tetani* strains. Genome IDs of modern *C. tetani* strains are listed in Chapeton-Montes *et al*. (2019). Sequences with 80% or greater coverage of the *C. tetani* E88 query sequences were aligned with MUSCLE v3.8.31, and RAxML (v8.2.4) trees using the GTR+GAMMA model were created. Bootstrap values (based on 100 runs) are displayed for major clades. The tree is highlighted as follows: GranCanaria-Tooth008 (dark grey) and clades 1 (yellow), 2 (purple), and X (light grey). Source data are provided as a Source Data file.

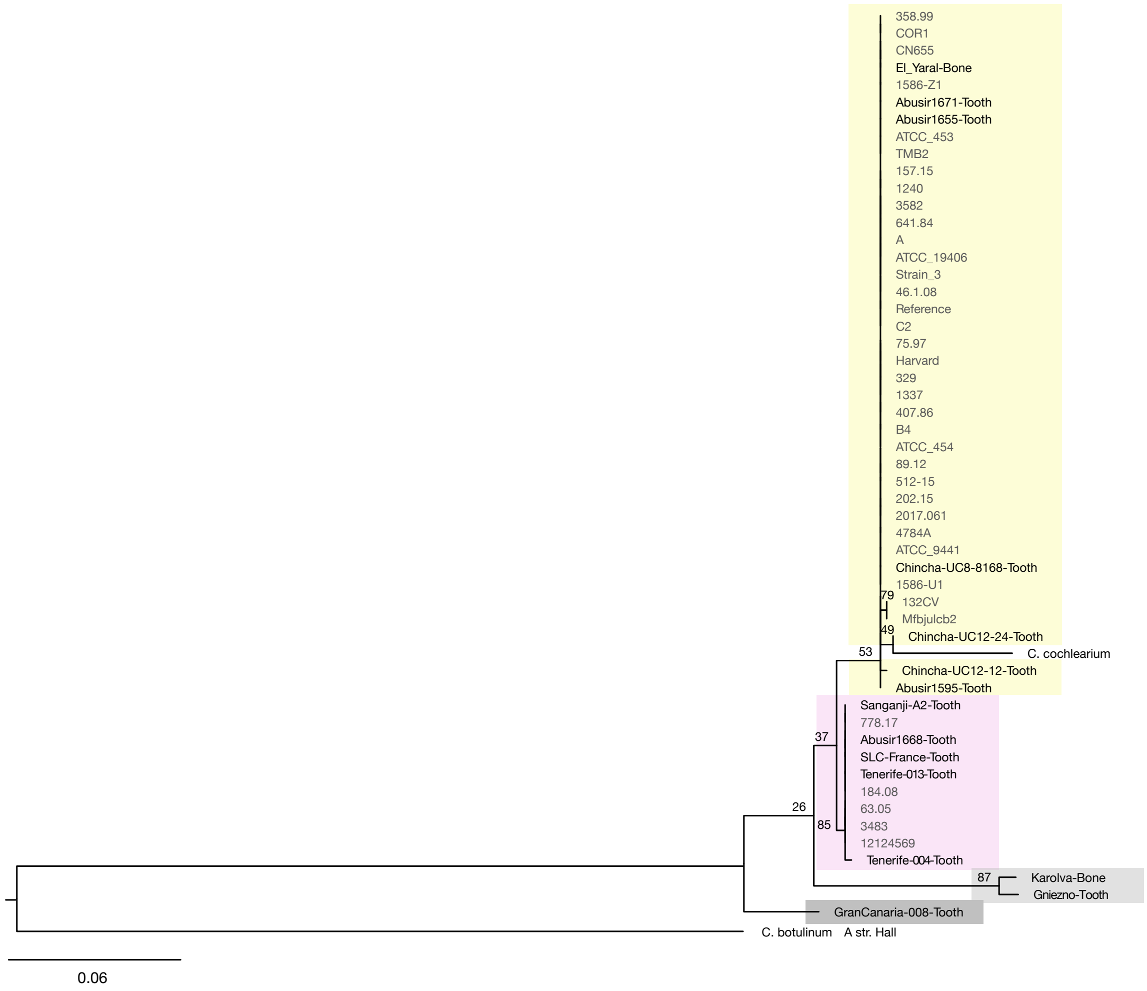

Supplementary Figure 13. Phylogenetic trees of *rpsG* coding sequences. Trees are based on a blastn search with *Clostridium tetani* E88 sequences. The phylogeny is based on a multiple alignment of *rpsG* (AE015927.1:c2752268-2751801) sequences identified from aDNA *C. tetani* contigs and modern *C. tetani* strains. Genome IDs of modern *C. tetani* strains are listed in Chapeton-Montes *et al*. (2019). Sequences with 80% or greater coverage of the *C. tetani* E88 query sequences were aligned with MUSCLE v3.8.31, and RAxML (v8.2.4) trees using the GTR+GAMMA model were created. Bootstrap values (based on 100 runs) are displayed for major clades. The tree is highlighted as follows: GranCanaria-Tooth008 (dark grey) and clades 1 (yellow), 2 (purple), and X (light grey). Source data are provided as a Source Data file.

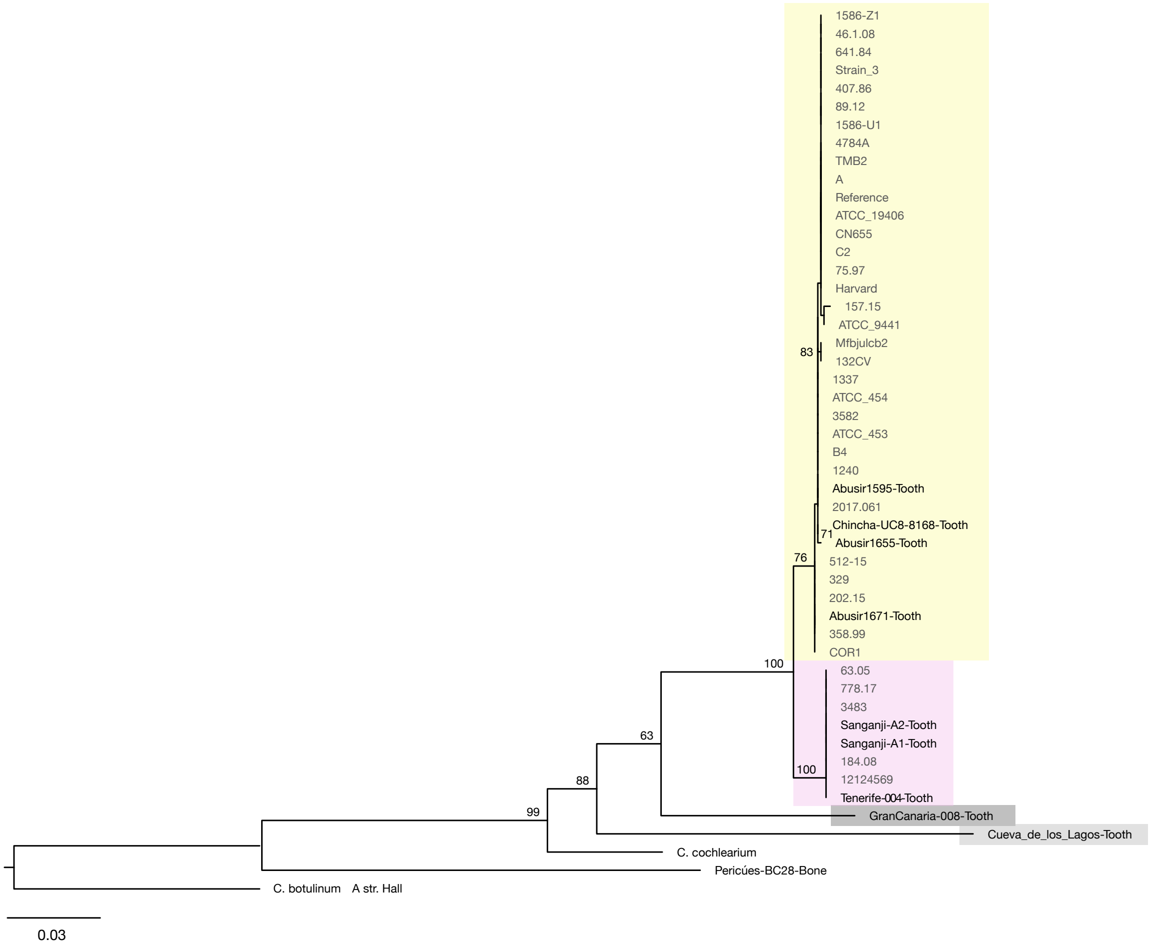

Supplementary Figure 14. Phylogenetic trees of *recA* coding sequences. Trees are based on a blastn search with *Clostridium tetani* E88 sequences. The phylogeny is based on a multiple alignment of *recA* (AE015927.1:1383544-1384548) sequences identified from aDNA *C. tetani* contigs and modern *C. tetani* strains. Genome IDs of modern *C. tetani* strains are listed in Chapeton-Montes *et al*. (2019). Sequences with 80% or greater coverage of the *C. tetani* E88 query sequences were aligned with MUSCLE v3.8.31, and RAxML (v8.2.4) trees using the GTR+GAMMA model were created. Bootstrap values (based on 100 runs) are displayed for major clades. The tree is highlighted as follows: GranCanaria-Tooth008 (dark grey) and clades 1 (yellow), 2 (purple), and X (light grey). Source data are provided as a Source Data file.

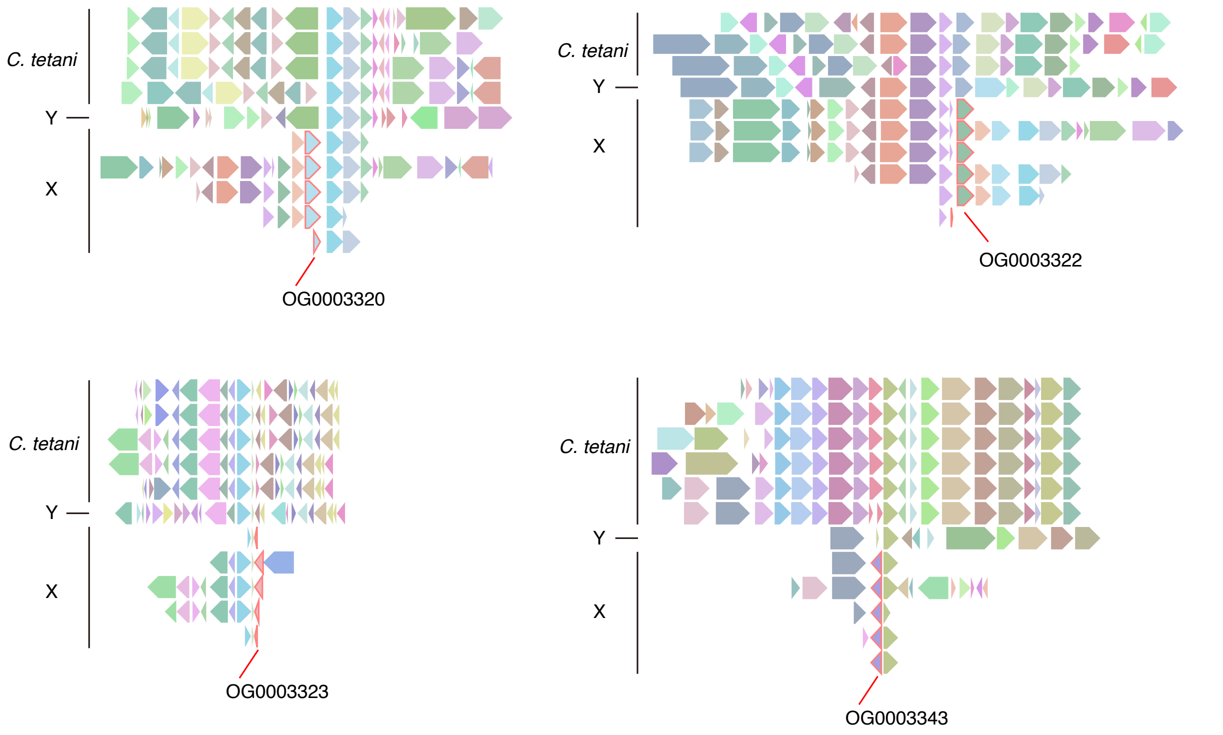

Closest BLAST matches:

OG0003320 - MYG1 family protein in *Lutibacter* sp. B2 (MBF8984673.1), 79.8 % identity
OG0003322 - EFR1 family ferrodoxin in *Desulforamulus reducens* (WP_011878543.1), 79.8 % identity
OG0003323 - SWIM zinc finger family protein in *Anaerophilus nitritogenes* (WP_129596215.1), 83.6 %ID
OG0003343 - No detected homologs

Supplementary Figure 15. Unique gene neighborhood structures in clade X and Y acBins compared to modern reference *C. tetani* strains. Orthology-based clustering of all protein sequences from our dataset identified fourteen orthogroups (genes) unique to four or more clade X members and absent among all *C. tetani* genomes. The genomic context of four of these genes (labeled by their orthogroup ID) is shown above. The highlighted genes in red are unique to clade X. The genes surrounding these X-specific genes are conserved in other *C. tetani* genomes. Gene neighborhood visualizations produced using AnnoView v1.0 (annoview.uwaterloo.ca).

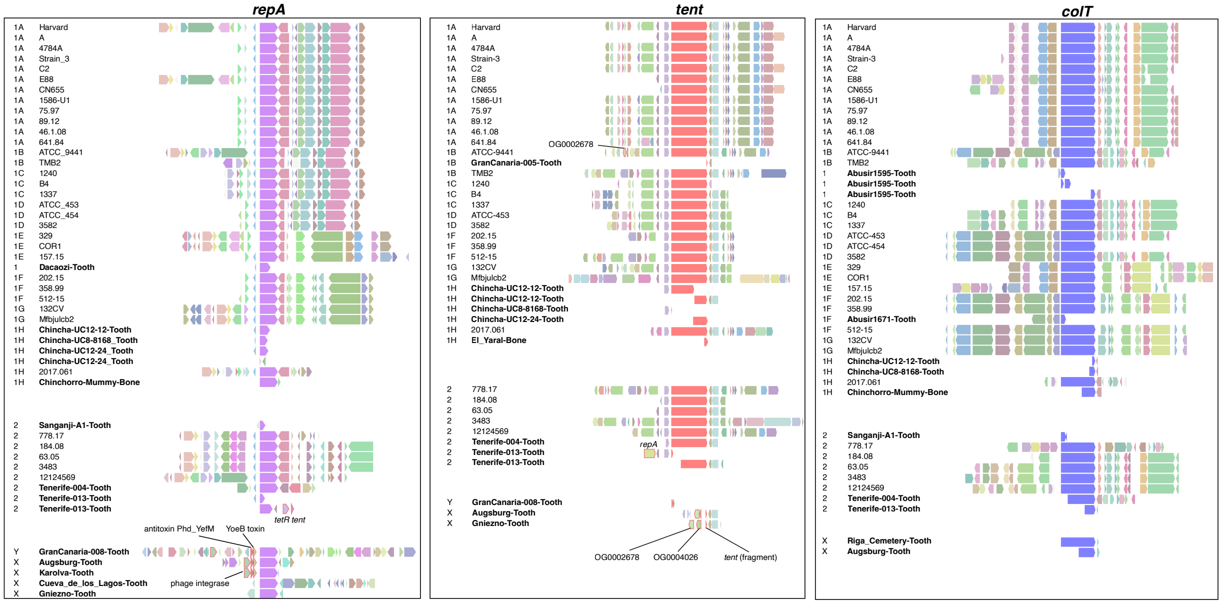

Supplementary Figure 16. Gene neighborhoods surrounding the *repA*, *colT, and tent* genes in select acBins and reference *C. tetani* genomes. Only assembled gene clusters with one or more genes surrounding the center gene were selected for display. Genes are colored uniquely based on their orthogroup membership as determined by OrthoFinder predictions. Gene clusters from *C. tetani* lineage 1, *C. tetani* lineage 2, and *Clostridium* sp. X and Y are grouped separately. In general, gene clusters from *C. tetani* lineage 1 and 2 share similarities, and those from *Clostridium* sp. X and Y contain patterns that are unique from *C. tetani*. On the left acBins names are bolded. Gene neighborhood visualizations produced using AnnoView v1.0 (annoview.uwaterloo.ca).

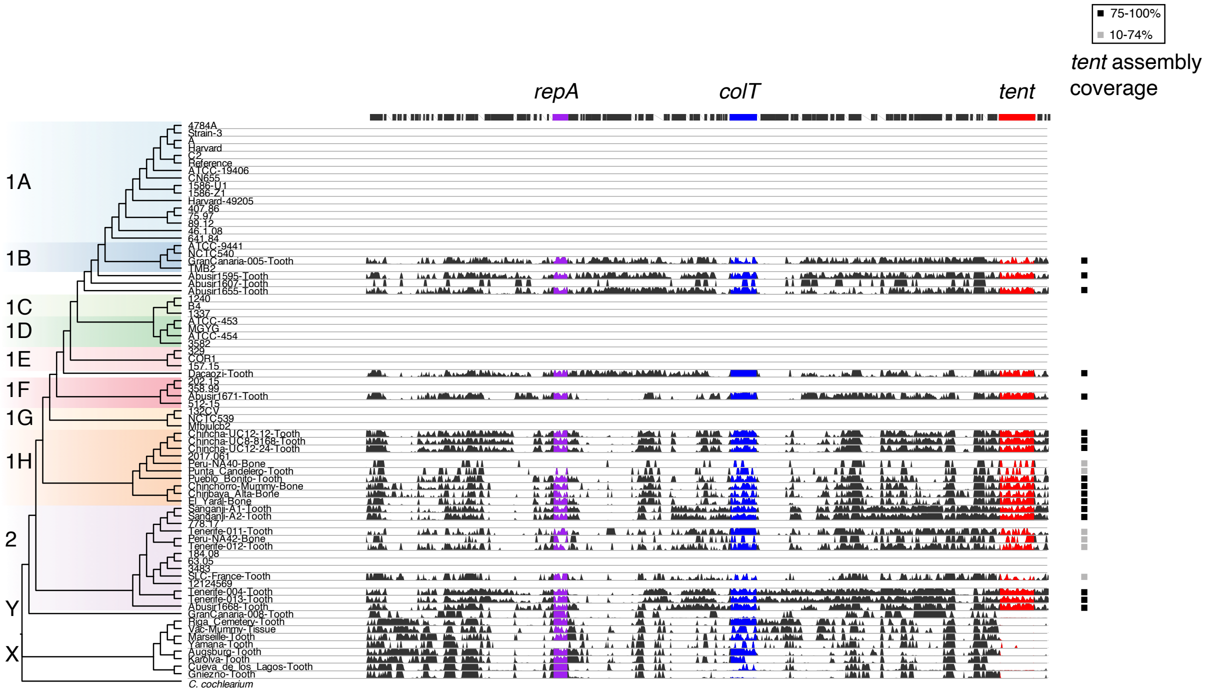

Supplementary Figure 17. Mapped read coverage to the reference *C. tetani* E88 plasmid and *tent* genes. Visualization of mapped read coverage to the *C. tetani* E88 plasmid. The genes (*tent*, *colT,* and *repA*) are coloured red, blue and purple, respectively. The plot reveals a relative lack of coverage in clade X acBins across the *tent* gene. Coverage values were capped to the 80th percentile to prevent high coverage regions from obscuring other regions. Genes were plotted as black bars using RefSeq annotations. On the right of the plot, the boxes indicate the acBins for which *tent* sequences were assembled with high coverage (black) or partially (gray) as described in the Methods.

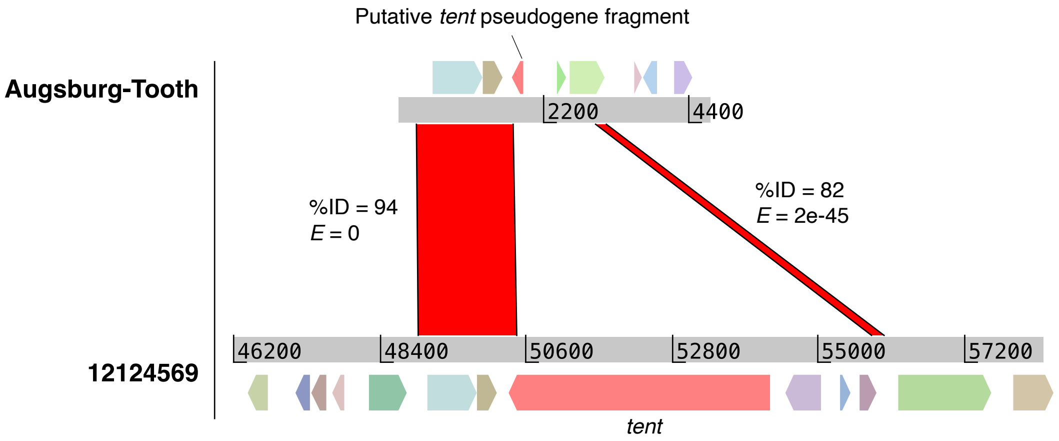

Supplementary Figure 18. Identification of a putative deletion event resulting in a possible *tent* pseudogene in clade X acBins. Two clade X acBins possess an assembled contig containing a *tent* fragment as shown in Figure 3b. A BLASTN comparison of this contig from Augsburg-Tooth against a representative *tent* locus from strain 12124569 reveals two regions of homology that flank the *tent* gene in strain 12124569. This indicates a possible rearrangement or deletion that has resulted in the removal of the *tent* gene and some adjacent sequence in Augsburg-Tooth.

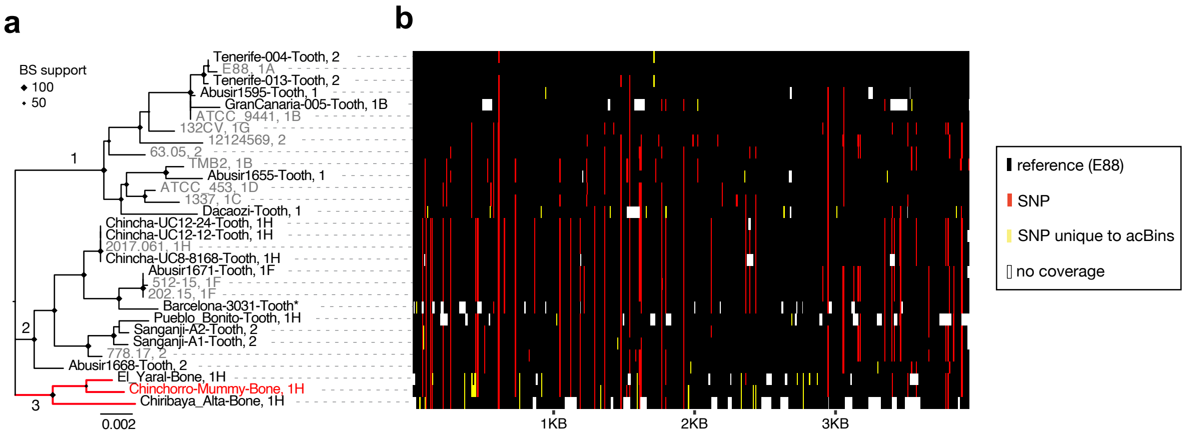

Supplementary Figure 19. Reconstructed *tent* sequences from ancient DNA samples display unique mutational profiles from modern sequences. Maximum-likelihood phylogenetic tree of *tent* genes (**a**) and visualization of SNP profiles relative to the E88 reference sequence (**b**). The visualization on the right plots the SNP profiles for each sequence, with yellow positions representing SNPs found uniquely in *tent* sequences from ancient DNA samples, red positions indicating SNPs also found in modern *tent* sequences, and black positions indicating identity to the reference E88 *tent* sequence. Gaps are colored white. The plot was generated by loading the *tent* MSA into R v4.1.0 with the Biostrings library v2.62.0, converting the MSA to a data matrix using a custom script (see Github repository) and plotted as a tile plot using the ggplot2 library v3.3.5. Source data are provided as a Source Data file.

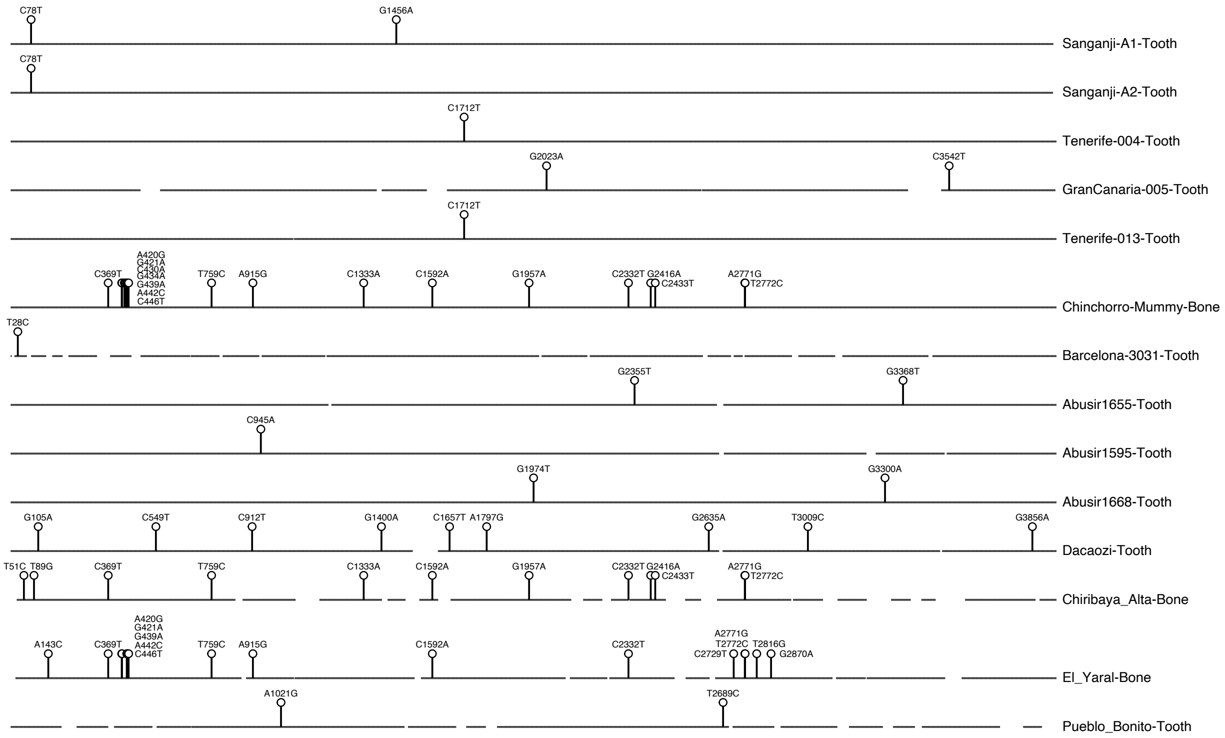

Supplementary Figure 20. Novel nucleotide substitutions in *tent* genes recovered from ancient samples that have not been identified in modern *tent* genes. Each sequence is depicted in their 5'-3' orientation. Horizontal lines indicate sequences that are identical to the E88 reference *tent* sequence, and missing lines indicate gaps (regions with no coverage). All SNPs are listed with numbering relative to the E88 reference *tent* sequence. Source data are provided as a Source Data file.

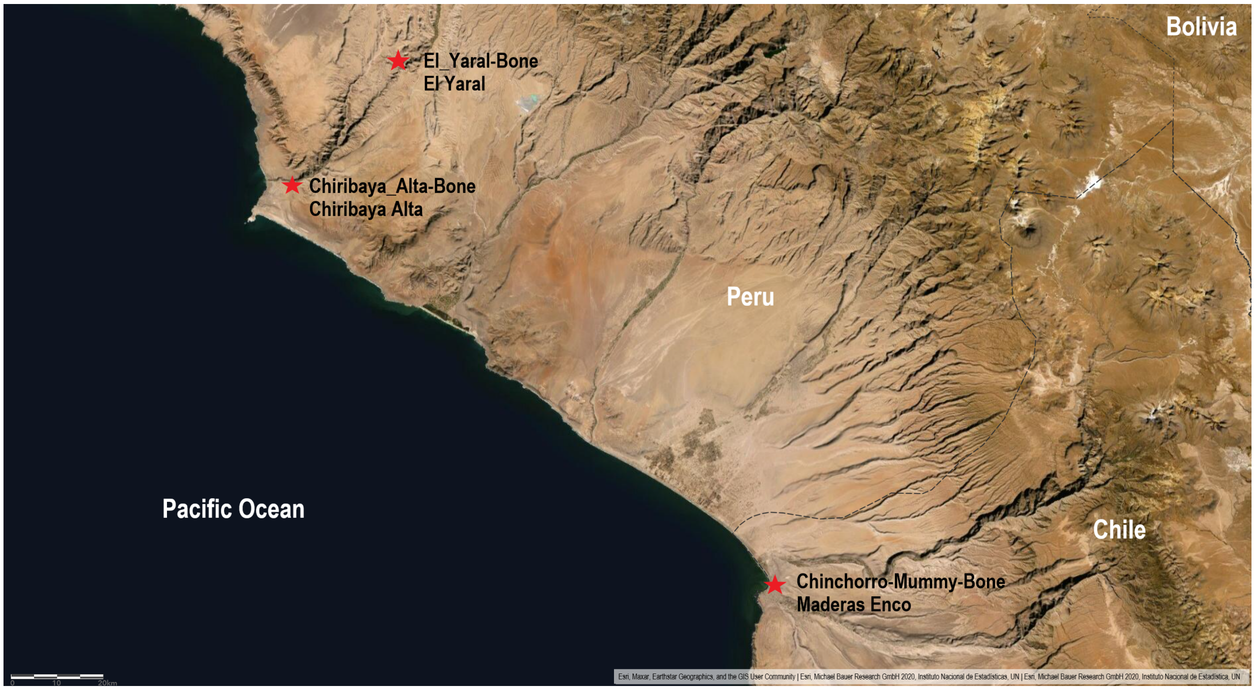

Supplementary Figure 21. Geographic location of three samples [Chinchorro-Mummy-Bone (SAMEA3486783), El_Yaral-Bone (SAMN02727821), and Chiribaya_Alta-Bone (SAMN02727818)] associated with subgroup '2' *tent* variants. This map was made by the authors using ArcGIS online, with the World Imagery (WGS84) basemap credited to: Esri, Maxar, Earthstar Geographics, and the GIS User Community; as well as the Chile Country Boundary 2020 and Bolivia Country Boundary 2020 layers, both credited to: Esri, Michael Bauer Research GmbH 2020, Instituto Nacional de Estadísticas, UN.

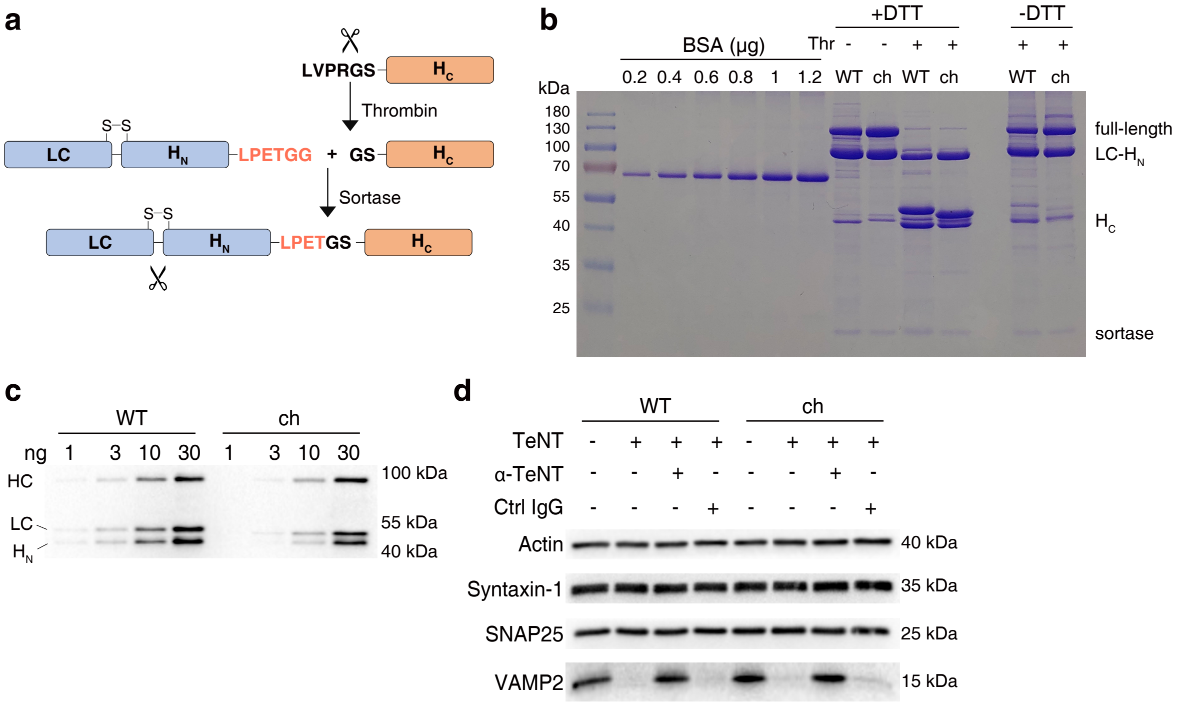

Supplementary Figure 22. Antiserum of TeNT neutralized TeNT and TeNT/Chinchorro with similar efficiency.
(**a**) The schematic drawing of sortase ligation method.
(**b**) Sortase ligation reaction mixtures were analyzed by SDS-PAGE and Coomassie blue staining. Thr, Thrombin.
(**c**) Rabbit antiserum of TeNT recognized full-length activated TeNT and TeNT/Chinchorro ("ch") with similar sensitivity by using western blot analysis.
(**d**) Cultured rat cortical neurons were exposed to 25 pM full-length toxins together with antiserum (α-TeNT, 1:1000) or control non-immunized serum (Ctrl IgG, 1:1000) for 12 hrs. Cell lysates were analyzed by immunoblot. For (b-d), the images shown are representatives of 3 independent experiments. Source data are provided as a Source Data file.
