# Supplementary material for: Ancient Clostridium DNA and variants of tetanus neurotoxins associated with human archaeological remains
Source: Nat Commun. 2023 Sep 6;14:5475. doi: 10.1038/s41467-023-41174-0 (PMC10482840; doi:10.1038/s41467-023-41174-0)
Supplement: Supplementary file 3 — Description of Additional Supplementary Files [file 41467_2023_41174_MOESM3_ESM.pdf]

Description of Additional Supplementary Files:

Supplementary Data 1: Metadata for SRA sequencing runs associated with high *C. tetani* abundance predicted using the SRA taxonomy analysis tool.

Supplementary Data 2: Metadata and bioinformatic annotations for BioSamples containing ancient human DNA and *C. tetani*. Metadata includes SRA-derived information as well as newly added annotations based on analysis of acBins.

Supplementary Data 3: Identified archaeal and bacterial species in *C. tetani*-containing human aDNA samples. Total counts for each biosample are derived from their respective SRA taxonomic profiles.

Supplementary Data 4: Species identified in 38 BioSamples and their environmental associations based on known literature. All species listed were detected with greater than 2% abundance of the microbial community based on the SRA STAT results.

Supplementary Data 5: Estimated abundance (of microbial community) of environment-specific microbes in the bacterial and archaeal fraction of 38 BioSamples. Environment-specific microbes were defined as shown in Supplementary Data 4.

Supplementary Data 6: Kaiju taxonomic classification summary at the species level of assembled samples (contig count).

Supplementary Data 7: Statistics for assembled contigs assigned to *C. tetani* after blast-validation.

Supplementary Data 8: CheckM analysis of all 38 acBin BLAST-verified *C. tetani* contigs.

Supplementary Data 9: MapDamage analysis of acBin contig sets. Contigs were BLAST-validated as mapping to *C. tetani* and contigs encoding rRNAs and tRNAs were removed.

Supplementary Data 10: PyDamage analysis of acBin contig sets. Contigs were BLAST-validated as mapping to *C. tetani* and contigs encoding rRNAs and tRNAs were removed.

Supplementary Data 11: Pairwise average nucleotide identity values for a *C. tetani* bin from a human gut sample (SRR10479805) to acBins and modern genomes from related *Clostridium* species.

Supplementary Data 12: Pairwise average nucleotide identity values for *Clostridium* sp. clade X acBins and modern genomes from related *Clostridium* species.

Supplementary Data 13: Pairwise average nucleotide identity values for lineage Y acBin (GranCanaria008-Tooth) and modern genomes from related *Clostridium* species.

Supplementary Data 14: Orthogroups of all modern *C. tetani* genomes and all acBins. Any orthogroup without at least one protein on a blast-validated contig and/or without at least one full length protein has been removed.

Supplementary Data 15: *C. tetani* orthogroups that contain at least one *C. tetani* E88 plasmid protein and their frequency in the other genomes/acBins. Protein descriptions are listed for orthogroups with >=90% frequency in high-completeness acBins.

Supplementary Data 16: *C. tetani* orthogroups that contain at least one *C. tetani* E88 sporulation-related protein and their frequency in the other genomes/acBins.

Supplementary Data 17: Per-sample coverage statistics and correlations for the *C. tetani* chromosome, plasmid, *repA*, *colT* gene, and *tent* gene.

Supplementary Data 18: Comparison of assembled *tent* sequences from ancient DNA samples with modern *tent* sequences.

Supplementary Data 19: Detected SNPs in *tent* sequences from acBins and modern *C. tetani* strains relative to *tent*/E88 reference.

Supplementary Data 20: *tent* variants detected in ancient samples using the Octopus variant caller. See VCF file format for header information.

Supplementary Data 21: Analysis of SNPs present in *tent* sequences from Chinchorro_Mummy_bone, El_Yaral_Bone, and Chibaya_AltaBone.

Supplementary Data 22: FASTQ processing statistics for ancient DNA samples with high predicted *C. tetani* content.

Supplementary Data 23: Genomes of *C. tetani* strains downloaded from the NCBI database and associated metadata.
