# Supplementary material for: Ancient Clostridium DNA and variants of tetanus neurotoxins associated with human archaeological remains
Source: Nat Commun. 2023 Sep 6;14:5475. doi: 10.1038/s41467-023-41174-0 (PMC10482840; doi:10.1038/s41467-023-41174-0)
Supplement: Supplementary file 5 — Reporting Summary [file 41467_2023_41174_MOESM5_ESM.pdf]

nature portfolio

Corresponding author(s): Andrew C. Doxey, Min Dong, Gabriel Renaud

Last updated by author(s): Aug 18, 2023

Reporting Summary

Nature Portfolio wishes to improve the reproducibility of the work that we publish. This form provides structure for consistency and transparency in reporting. For further information on Nature Portfolio policies, see our Editorial Policies and the Editorial Policy Checklist.

Statistics

For all statistical analyses, confirm that the following items are present in the figure legend, table legend, main text, or Methods section.

n/a	Confirmed	
☐	☒	The exact sample size (*n*) for each experimental group/condition, given as a discrete number and unit of measurement
☐	☒	A statement on whether measurements were taken from distinct samples or whether the same sample was measured repeatedly
☐	☒	The statistical test(s) used AND whether they are one- or two-sided<br>*Only common tests should be described solely by name; describe more complex techniques in the Methods section.*
☐	☒	A description of all covariates tested
☐	☒	A description of any assumptions or corrections, such as tests of normality and adjustment for multiple comparisons
☐	☒	A full description of the statistical parameters including central tendency (e.g. means) or other basic estimates (e.g. regression coefficient) AND variation (e.g. standard deviation) or associated estimates of uncertainty (e.g. confidence intervals)
☐	☒	For null hypothesis testing, the test statistic (e.g. *F*, *t*, *r*) with confidence intervals, effect sizes, degrees of freedom and *P* value noted<br>*Give P values as exact values whenever suitable.*
☒	☐	For Bayesian analysis, information on the choice of priors and Markov chain Monte Carlo settings
☒	☐	For hierarchical and complex designs, identification of the appropriate level for tests and full reporting of outcomes
☐	☒	Estimates of effect sizes (e.g. Cohen's *d*, Pearson's *r*), indicating how they were calculated

Our web collection on statistics for biologists contains articles on many of the points above.

Software and code

Policy information about availability of computer code

Data collection	The following tools were used for data collection: Google Cloud SDK v330.0.0, and sra-toolkit v2.9.6. Custom code used for data collection is provided here: https://github.com/harohodg/aDNA-tetanus-analysis
Data analysis	The following tools were used for data analysis: R v4.0.4 and v4.1.0, seqtk v1.3, fastp v0.20.1, MEGAHIT v1.2.9, Kaiju v1.7.4, Blast+ v2.12.0, Barrnap v0.9, Aragorn v1.2.36, CheckM v1.0.18, samtools v1.12 and v1.15.1, fastANI v1.33, seqinr v4.2-16, sra-toolkit v2.9.6, Bowtie2 v2.4.2, matplotlib v3.3.2, Python v3.8.5, Rsamtools library v2.8.0, ggplot2 v3.3.5, Biostrings 2.62.0, leeHom v1.2.15, BWA v0.7.17-r1188, mapDamage v2.2.1, pyDamage v0.70, Snippy v4.6.0, FastTree v2.1.10, RaxML v8.2.12, Gubbins v3.3, Phandango v.1.3.0, Harvest v1.2, Parsnp v1.2, Gingr v1.3, prodigal v2.6.2, Orthofinder v2.5.4, AnnoView v1.0, bcftools v1.12, htslib v1.12, Octopus variant caller v0.7.4, MAFFT v7.4.80, SWISSMODEL server, PyMOL v2.4.1, Jalview v2.9.0b2, PhyML v3.1. Custom code used for data data analysis is provided here: https://github.com/harohodg/aDNA-tetanus-analysis

For manuscripts utilizing custom algorithms or software that are central to the research but not yet described in published literature, software must be made available to editors and reviewers. We strongly encourage code deposition in a community repository (e.g. GitHub). See the Nature Portfolio guidelines for submitting code & software for further information.

Data

Policy information about availability of data

All manuscripts must include a data availability statement. This statement should provide the following information, where applicable:

- Accession codes, unique identifiers, or web links for publicly available datasets
- A description of any restrictions on data availability
- For clinical datasets or third party data, please ensure that the statement adheres to our policy

Source data are provided with this paper. Raw genomic data reported in this study is available from the NCBI sequence read archive. Accession numbers for all BioSamples and sequencing runs used are listed in Supplementary Data 1 and 2. Processed data generated in this study are provided in the Supplementary Data and Source Data files. Additional large datasets generated by this study have been deposited in FigShare under the following accession numbers: https://doi.org/10.6084/m9.figshare.21498198, https://doi.org/10.6084/m9.figshare.21498222, https://doi.org/10.6084/m9.figshare.21498330, https://doi.org/10.6084/m9.figshare.21652340, and https://doi.org/10.6084/m9.figshare.23804106.

Research involving human participants, their data, or biological material

Policy information about studies with human participants or human data. See also policy information about sex, gender (identity/presentation), and sexual orientation and race, ethnicity and racism.

Reporting on sex and gender	As our study focuses on microbial (Clostridium tetani related DNA) obtained from archaeological samples, this section does not apply.
Reporting on race, ethnicity, or other socially relevant groupings	See above.
Population characteristics	See above.
Recruitment	See above.
Ethics oversight	See above.

Note that full information on the approval of the study protocol must also be provided in the manuscript.

Field-specific reporting

Please select the one below that is the best fit for your research. If you are not sure, read the appropriate sections before making your selection.

[x] Life sciences [ ] Behavioural & social sciences [ ] Ecological, evolutionary & environmental sciences

For a reference copy of the document with all sections, see nature.com/documents/nr-reporting-summary-flat.pdf

Life sciences study design

All studies must disclose on these points even when the disclosure is negative.

Sample size	Bioinformatic analysis: N = 136 sequencing runs from the NCBI SRA database were selected for analysis as these surpassed a C. tetani k-mer threshold of 23,000. We selected this threshold because the datasets above this threshold had a C. tetani DNA content that was sufficient to enable our intended analyses (e.g., genome reconstruction). The threshold chosen also successfully identified 28 positive control datasets (runs associated with C. tetani genome projects). Of these 136 sequencing runs, 76 were selected for analysis as these were associated with ancient DNA samples (N = 38 unique BioSamples). The sample size (38 unique BioSamples) was sufficient for our analysis because it allowed us to analyze a diversity of samples in terms of their spatial and temporal origin. As the main finding of our study is the discovery of C. tetani in aDNA samples, including as many viable samples as possible was important to reinforce our claims by providing additional biologically independent aDNA samples that demonstrate the presence of C. tetani DNA. As described above, including additional samples below the threshold cutoff would have been unlikely to yield a substantial number of additional C. tetani containing aDNA samples at a quantity sufficient to enable our genomic analyses, and even if such samples could be included, this would not alter the conclusions reached in our manuscript.<br><br>Mouse bioassays: Our in vivo tetanus assay was designed to detect potential loss of function or gain of function of the TeNT/Chinchorro protein. Based on our experience with IM injection of TeNT/BoNT toxins, 3 mice per group is sufficient to show significant differences of more than 50% in toxin potency [Thaker H, Zhang J, Miyashita S-I, Cristofaro V, Park S, Hashemi Gheinani A, et al. (2021) Knockin mouse models demonstrate differential contributions of synaptotagmin-1 and -2 as receptors for botulinum neurotoxins. PLoS Pathog 17(10): e1009994. https://doi.org/10.1371/journal.ppat.1009994].
Data exclusions	In constructing the SNP-based phylogenomic tree including ancient C. tetani genomes, five samples were excluded. Five acMAGs were omitted due to extremely low (<1%) genome coverage, which could result in phylogenetic artifacts. These five samples were: SAMEA103957995, SAMEA103971604, SAMEA3486793, SAMEA104402285, and SAMEA3937653.<br><br>In the reduced phylogenetic tree of Figure 2c, only 11 acBins were included as these passed the thresholds of Parsnp (as described in manuscript).

In Supplementary Figure 3, Peru-NA42-Bone was not included in (a-d) as it contains a mix of pre- and post-capture sequences.

Six samples were removed from mtDNA mapdamage plots as they contained an insufficient number of reads mapping to the human mitochondrial genome (described in Supplementary Figure 5 legend).

Samples with full or partial UDG treatment were also removed from several analyses of damage rates as this is a known confounding variable (described in Supplementary Figure 6 legend).

In Supplementary Figure 8, genomes with gap content of 90% or greater were excluded.

Replication

All attempts at reproducibility were successful. All source code used has been provided open-source in github repositories, which facilitates reproducibility of all data analyses.

Randomization

Bioinformatic analysis: Randomization was not needed in any statistical analyses presented in our work. In the analysis of damage rates, we accounted for other co-variates that could influence damage rates by subdividing our dataset into different metadata categories and performing statistical comparisons between these groups. Specifically, we compared damage rates between UDG treated (partial, full) and untreated samples, examined the influence of sample age, and capture method. The influence of these factors on damage rates is described in our manuscript.

Experimental analyses: Mice were randomly assigned to groups by an investigator that was blind to the health and behavioral status of the mice.

Blinding

Bioinformatic analysis: Blinding was not possible during initial group allocation because all samples were manually assigned their group category (aDNA, modern, etc.) by the investigators through examination of sample metadata and associated literature. However, blinding was not relevant in the analyses of this study as all samples were analyzed in the same manner by our computational pipeline with the exception of sample restrictions in some analyses due to quality thresholds and covariate information.

Animal experiments were scored by an investigator blind to the identity of the toxin that was administered.

Reporting for specific materials, systems and methods

We require information from authors about some types of materials, experimental systems and methods used in many studies. Here, indicate whether each material, system or method listed is relevant to your study. If you are not sure if a list item applies to your research, read the appropriate section before selecting a response.

Materials & experimental systems

n/a	Involved in the study	
☐	☒	Antibodies
☒	☐	Eukaryotic cell lines
☒	☐	Palaeontology and archaeology
☐	☒	Animals and other organisms
☒	☐	Clinical data
☐	☒	Dual use research of concern
☒	☐	Plants

Methods

n/a	Involved in the study	
☒	☐	ChIP-seq
☒	☐	Flow cytometry
☒	☐	MRI-based neuroimaging

Antibodies

Antibodies used

Syntaxin-1 1:2000 Synaptic Systems 110011
SNAP25 1:2000 Synaptic Systems 111008
VAMP1/2/3 1:1000 Synaptic Systems 104102
β- Actin 1:2000 Sigma-Aldrich A5441
Goat anti-Rabbit IgG (H+L) Secondary Antibody, HRP 1:2000 Invitrogen 65-6120
Goat anti Mouse IgG (H/L):HRP 1:2000 Bio-Rad STAR207P

Validation

Syntaxin-1 Synaptic Systems 110011 was validated by IP, which included complexes with synaptobrevin and SNAP25, and it has also been WB validated by more than 37 papers. Please see: https://sysy.com/product/110011

SNAP25 Synaptic Systems 111008 (clone71.1) was K.O. validated. Please see: PMID 31794878.

VAMP1/2/3 Synaptic Systems 104102 was validated by IP (PMID: 31940485), which included other SNARE complex proteins. It has also been WB validated by multiple studies. Please see: https://sysy.com/product/104102

β- Actin Sigma-Aldrich A5441 (clone AC-15) was K.O. validated. Please see: https://www.abcam.com/products/primary-antibodies/

beta-actin-antibody-ac-15-ab6276.html

Animals and other research organisms

Policy information about studies involving animals; ARRIVE guidelines recommended for reporting animal research, and Sex and Gender in Research

Laboratory animals	Mice (CD-1 strain, female, purchased from Envigo, 6-7 weeks old, 25–28 g, n=3 per group, 9 total).<br><br>Mice were housed in a specific-pathogen free mouse facility on a 12hr light and dark cycle with ad lib access to food and water. Mice were maintained at 22°C+/- 2°C, 35-70% humidity.<br><br>For culture of rat embryonic cortical neurons, timed-mating pregnant rats were purchased from Envigo (Sprague Dawley) and embryos used were E18. Rats were not kept in our facility. We do not track the number of embryos used for culture, and that is not regulated since the animals are not born/weaned. The embryo will be both male and female, and the rats are all female.
Wild animals	The study did not involve wild animals.
Reporting on sex	In vitro findings apply to both sexes; in vivo findings apply to female mice. There are no findings indicating that tetanus sensitivity varies between sex and both male and female humans are susceptible to tetanus. Drawing from our years of experience in conducting mouse bioassays within our laboratory, we have observed a trend where larger or older mice seem to exhibit greater tolerance to TeNT. CD-1 mice are sold by weight and not developmental stage, therefore it may be difficult to ensure these confounds are adjusted for. The major purpose of this assay was to confirm that tetanus mutants remain functional rather than determining host differences in tetanus sensitivity. We choose female mice, because they do not engage in aggressive behaviors, and their sensitivity is well characterized in our laboratory, therefore we are able to reduce the number of animals used.
Field-collected samples	The study did not involve samples collected from the field.
Ethics oversight	All animal studies were approved by the Boston Children's Hospital Institutional Animal Care and Use Committee (Protocol Number: 18-10-3794R).

Note that full information on the approval of the study protocol must also be provided in the manuscript.

Dual use research of concern

Policy information about dual use research of concern

Hazards

Could the accidental, deliberate or reckless misuse of agents or technologies generated in the work, or the application of information presented in the manuscript, pose a threat to:

No	Yes	
☒	☐	Public health
☒	☐	National security
☒	☐	Crops and/or livestock
☒	☐	Ecosystems
☒	☐	Any other significant area

Experiments of concern

Does the work involve any of these experiments of concern:

No	Yes	
☒	☐	Demonstrate how to render a vaccine ineffective
☒	☐	Confer resistance to therapeutically useful antibiotics or antiviral agents
☒	☐	Enhance the virulence of a pathogen or render a nonpathogen virulent
☒	☐	Increase transmissibility of a pathogen
☒	☐	Alter the host range of a pathogen
☒	☐	Enable evasion of diagnostic/detection modalities
☒	☐	Enable the weaponization of a biological agent or toxin
☒	☐	Any other potentially harmful combination of experiments and agents
